# *Bacteroides acidifaciens* and its derived extracellular vesicles improve DSS-induced colitis

**DOI:** 10.3389/fmicb.2023.1304232

**Published:** 2023-11-30

**Authors:** Cihua Zheng, Yuchun Zhong, Jian Xie, Zhuoya Wang, Wenming Zhang, Yiming Pi, Wenjun Zhang, Li Liu, Jun Luo, Wei Xu

**Affiliations:** ^1^Department of General Surgery, The Second Affiliated Hospital of Nanchang University, Nanchang, Jiangxi, China; ^2^Department of Rehabilitation Medicine, The Second Affiliated Hospital of Nanchang University, Nanchang, Jiangxi, China; ^3^The Institute of Translational Medicine, The Second Affiliated Hospital of Nanchang University, Nanchang University, Nanchang, Jiangxi, China; ^4^Graduate School of Jiangxi University of Chinese Medicine, Nanchang, China

**Keywords:** inflammatory bowel disease, *Bacteroides acidifaciens*, extracellular vesicles, gut microbiota, intestinal mucosal barrier

## Abstract

**Introduction:**

“Probiotic therapy” to regulate gut microbiota and intervene in intestinal diseases such as inflammatory bowel disease (IBD) has become a research hotspot. *Bacteroides acidifaciens*, as a new generation of probiotics, has shown beneficial effects on various diseases.

**Methods:**

In this study, we utilized a mouse colitis model induced by dextran sodium sulfate (DSS) to investigate how B. acidifaciens positively affects IBD. We evaluated the effects of*B. acidifaciens*, fecal microbiota transplantation, and bacterial extracellular vesicles (EVs) on DSS-induced colitis in mice. We monitored the phenotype of mouse colitis, detected serum inflammatory factors using ELISA, evaluated intestinal mucosal barrier function using Western blotting and tissue staining, evaluated gut microbiota using 16S rRNA sequencing, and analyzed differences in EVs protein composition derived from *B. acidifaciens* using proteomics to explore how *B. acidifaciens* has a positive impact on mouse colitis.

**Results:**

We confirmed that *B. acidifaciens* has a protective effect on colitis, including alleviating the colitis phenotype, reducing inflammatory response, and improving intestinal barrier function, accompanied by an increase in the relative abundance of *B. acidifaciens* and Ruminococcus callidus but a decrease in the relative abundance of *B. fragilis*. Further fecal bacterial transplantation or fecal filtrate transplantation confirmed the protective effect of eosinophil-regulated gut microbiota and metabolites on DSS-induced colitis. Finally, we validated that EVs derived from *B. acidifaciens* contain rich functional proteins that can contribute to the relief of colitis.

**Conclusion:**

Therefore, *B. acidifaciens* and its derived EVs can alleviate DSS-induced colitis by reducing mucosal damage to colon tissue, reducing inflammatory response, promoting mucosal barrier repair, restoring gut microbiota diversity, and restoring gut microbiota balance in mice. The results of this study provide a theoretical basis for the preclinical application of the new generation of probiotics.

## 1 Introduction

Inflammatory bowel disease (IBD) is a chronic disease with unclear pathogenesis and a repeated course and is mainly divided into Crohn’s disease and ulcerative colitis (Jakubczyk et al., 2020; Santana et al., 2022). The basic characteristics of IBD are abdominal pain and diarrhea, which can be complicated with colon stenosis and obstruction and tissue fibrosis and even induce colon cancer (Ullman and Itzkowitz, 2011). Since 1950, the number of patients in the western world, represented by Europe and America, has gradually increased, and recent epidemiological surveys show that the number of patients in eastern countries has also begun to increase rapidly (Molodecky et al., 2012). IBD has become a global disease. At present, although there are many treatments for IBD, such as hormones, immunosuppressants, biological agents, small molecule drugs and surgical treatments, the therapeutic effects are limited due to the unclear disease mechanism (Rogler et al., 2021).

Although the etiology of IBD is unknown, it is recognized that IBD is influenced by heredity, environment and intestinal microbiota, and it is characterized by a host immune response, intestinal barrier destruction and intestinal microbial community changes (Kudelka et al., 2020). The intestinal microbiota plays an important role in maintaining the intestinal mucosal barrier, stabilizing intestinal immune function and regulating intestinal metabolism (Li et al., 2021). However, intestinal microbiota imbalance or gut microbiota displacement can lead to intestinal inflammation and even tumors (Deng et al., 2022). Studies have shown that compared with healthy people, the ecological imbalance of IBD is characterized by a decrease in bacterial diversity or a change in composition (Franzosa et al., 2018). Therefore, it has become a research hotspot to intervene in intestinal diseases such as IBD by regulating intestinal microbiota. For example, “probiotic therapy,” in which enough live probiotics are supplemented to maintain the health of the host, has been widely used in the clinic and has achieved certain results (Fedorak et al., 2015; Tamaki et al., 2015).

Under physiological conditions, 90% of the intestinal microflora are composed of *Firmicutes* and *Bacteroides* (Eckburg et al., 2005). *Bacteroides acidifaciens* belongs to *Bacteroides*, a gram-negative anaerobic bacterium and one of the dominant symbiotic bacteria in the intestine (Momose et al., 2011). A study revealed that in a mouse model, *B. acidifaciens* has a protective effect on concanavalin A-induced liver injury (Wang et al., 2022). Nagahara et al. revealed that *B. acidifaciens* can increase the level of intestinal immunoglobulin A (IgA), protect the intestine from pathogen infection and relieve IBD (Nakajima et al., 2020). Another study showed that alginate oligosaccharide and its compound supplement may enhance immune function and improve intestinal microecological disorder by increasing the abundance of *B. acidifaciens* (Yan et al., 2023). Tamas Korcsmaros et al. revealed that bacterial extracellular vesicles (EVs) produced by the intestinal symbiotic bacteria *Bacteroides thetaiotaomicron* have potential therapeutic value in IBD (Gul et al., 2022). EVs are small, spherical double-layer structures (20∼400 nm) containing proteins, lipids, nucleic acids and some metabolites, which are secreted by gram-negative bacteria and some gram-positive bacteria (Kaparakis-Liaskos and Ferrero, 2015; Toyofuku et al., 2019). EVs are important cell communication carriers that participate in the exchange of biological information between prokaryotes and eukaryotes. EVs can pass through the aseptic mucous layer of the colon through different routes, enter the border intestinal epithelial cells, interact with mucosal immune cells and the intestinal vascular system, and promote its widespread and systemic spread (Chronopoulos and Kalluri, 2020; Liu J. H. et al., 2021; Chen et al., 2022). Previous studies have shown that EVs play an important role in regulating the intestinal microenvironment and host health (Díaz-Garrido et al., 2021; Liu L. et al., 2021). This indicates that bacterial EVs are a possible factor by which the intestinal microbiota alleviates diseases.

In this study, we used *B. acidifaciens* to interfere with dextran sulfate sodium (DSS)-induced colitis in mice, revealing its anti-inflammatory and barrier protection effects on enteritis mice. The effects of *B. acidifaciens*-mediated microorganisms and their metabolites on alleviating the symptoms of enteritis mice were confirmed by fecal bacteria transplantation (FMT) and aseptic fecal filtrate transplantation (FFT). Finally, we found that EVs of *B. acidifaciens* can inhibit inflammatory reactions, repair the intestinal mucosal barrier, and finally alleviate colitis in mice. Through proteomic analysis, it was confirmed that the functional proteins of *B. acidifaciens* and *B. acidifaciens*-EVs were different.

## 2 Materials and methods

### 2.1 Ethics statement

This study was approved by the Animal Experiment Ethics Committee of Nanchang University (NCULAE-20221031149).

### 2.2 Culture of bacteria

*Bacteroides acidifaciens* (BNCC353574, BeNa, Henan, China) was cultured on fastidious anaerobe broth (FAB, Cat# LA4550; Solarbio, Inc., China) or Columbia agar (Cat#HB8511; Qingdao Hope Bio-Technology, Co., Ltd., China) with 5% goat blood (Cat# TX0020; Solarbio, Inc., China) under anaerobic conditions at 37°C for 48–72 h (Wang et al., 2022). The bacterial concentration was calculated by counting the number of CFUs after plating on Columbia agar containing 5% goat blood.

### 2.3 Isolation and identification of EVs from *B. acidifaciens*

The conditioned medium containing *B. acidifaciens* was obtained as described above. The conditioned culture of *B. acidifaciens* was centrifuged at 15,000 × *g* at 4°C for 20 min and then filtered through a 0.45 μm sterile filter membrane to remove residual bacteria and debris. The filtered supernatant was centrifuged at 20,000 × *g* at 4°C for 40 min and then filtered through a 0.22 μm sterile filter membrane. The supernatant was transferred to 100 kDa Amicon Ultra15 filters at a concentration of 300–400 times. The concentrated solution was centrifuged at 120,000 × *g* at 4°C for 2 h to remove the supernatant, washed with sterile PBS, and centrifugation was repeated once to obtain EVs (Ma et al., 2022). As described, EVs were purified by OptiPrep (Cat# D1556, Sigma Aldrich, USA) gradient density centrifugation. Nanoparticle tracking analysis (NTA) method was used to detect the particle size distribution of EVs, and transmission electron microscopy (TEM) was used to observe the morphology of EVs.

### 2.4 Animal experiments

In this study, male C57BL/6J mice aged 6–8 weeks and weighing approximately 22–25 g were purchased from Tianqin Biotechnology Co., Ltd (Changsha, Hunan, China). Mice were reared at 22 ± 2°C for 1 week under an alternating light/dark cycle every 12 h. One week later, the mouse colitis model was induced by 2.5% dextran sodium sulfate (molecular mass 36–50 kDa, MP Biologicals, Solon, OH, USA) in a free drink. To study the therapeutic effect of *B. acidifaciens* on DSS-induced colitis, 24 mice were divided into three groups (*n* = 8): C group: free access to drinking water and food; M group: 2.5% DSS in a free drink for 7 days and daily oral administration of 0.1 ml saline for 10 days; TBA group: 2.5% DSS in a free drink for 7 days and daily oral administration of 0.1 ml 1 × 10^9^ CFU *B. acidifaciens* for 10 days.

To determine whether the therapeutic effect of *B. acidifaciens* on colitis works through metabolites, we performed FMT or FFT on DSS-induced mice (Wu et al., 2020). Twenty-four mice were treated with an antibiotic cocktail [ABX; metronidazole (1 g/L), neomycin sulfate (1 g/L), ampicillin (0.5 g/L), vancomycin (0.5 g/L)] for 7 days before fecal bacteria transplantation and then divided into 4 groups: M-FMT group: 2.5% DSS for 7 days and transplanted with fecal supernatant of the M group for 10 days; M-FFT group: 2.5% DSS for 7 days and transplanted with fecal supernatant filtrate of the M group for 10 days; BA-FMT group: 2.5% DSS for 7 days and transplanted with fecal supernatant of the *B. acidifaciens* treatment group for 10 days; BA-FFT group: 2.5% DSS for 7 days and transplanted with fecal supernatant filtrate of the *B. acidifaciens* treatment group for 10 days. After 7 days of ABX administration, 24 mouse fecal samples were anaerobic cultured on a Brain Heart Infusion Broth (BHI; Cat# HB8297-1; Qingdao Hope Bio-Technology, Co., Ltd., China) agar plate for 96 h to ensure the depletion of fecal microbiota. The feces of mice were collected and diluted with PBS (1:10), thoroughly homogenized, centrifuged at 500 × *g* for 1 min and collected the supernatant for FMT. The supernatant was filtered through a 0.22 μm filter membrane, and the filtrate was collected for FFT.

To study the therapeutic effect of EVs of *B. acidifaciens* on DSS-induced colitis, 24 mice were divided into 3 groups (*n* = 8): C group: free access to drinking water and food; M group: 2.5% DSS in a free drink for 7 days and daily oral administration of 0.1 ml saline for 10 days; TBA group: 2.5% DSS in a free drink for 7 days and daily oral administration of 0.1 ml EVs (50 μg/day) for 10 days. The weight of the mice was measured daily throughout the study. The disease activity index (DAI) evaluated the severity of colitis through weight loss, diarrhea of the stool, and the extent of blood in the feces (Ma et al., 2022).

### 2.5 H&E, AB/PAS, immunofluorescence and TUNEL staining

To evaluate the morphological changes in colon tissue in mice, the sample was fixed with 4% paraformaldehyde, embedded in paraffin, cut into 5–6 μm thick sections, rehydrated with xylene and degraded ethanol for 3–5 min, washed with PBS, and then stained with hematoxylin-eosin (H&E). To evaluate the number of goblet cells in the colon, we also stained fixed colon tissue sections with Alcian blue/periodic acid-Schiff staining (AB/PAS). To assess the expression of the colon barrier proteins ZO-1 (Cat# 21773-1-AP; Proteintech Group, Inc.; 1:100, China) and Occludin (Cat# 27260-1-AP; Proteintech Group, Inc.; 1:100, China), the tissue sections were stained with immunofluorescence. To assess the apoptosis of colon tissue cells in mice, fixed colon sections were stained with a terminal deoxynucleotidyl transferase-mediated dUTP nick end labeling (TUNEL) detection kit (Cat# T2196; Solarbio, Inc., China). Finally, the images were visualized by means of microscopy or fluorescence microscopy (Wu Z. et al., 2021).

### 2.6 Measurement of inflammatory cytokines and antioxidant indices in serum

To evaluate inflammation and oxidative stress in the serum of mice, the levels of the proinflammatory factors interleukin (IL)-1β (Cat# 2M-KMLJM211201m mouse; Camilo Biological, Nanjing, China), IL-6 (Cat# 2M-KMLJM219451m mouse; Camilo Biological, Nanjing, China), and tumor necrosis factor (TNF)-α (Cat# 2M-KMLJM220051m mouse; Camilo Biological, Nanjing, China) were determined according to the manufacturer’s protocol.

### 2.7 Western blotting analysis

Mice colon tissue sample proteins were extracted using cell lysis buffer (Cat# R0020; Solarbio, Inc., China) supplemented with phenylmethanesulfonyl fluoride (PMSF; Cat# P0100; Solarbio, Inc., China) and protease inhibitor cocktail (Cat# HY-K0022; Med Chem Express, Inc., China). A bicinchoninic acid protein assay kit (BCA; Cat# PC0020; Solarbio, Inc., China) was used to determine the protein concentration of the samples. Then, the proteins were detached with 8–10% polyacrylamide resolving gels, transferred to polyvinylidene fluoride membranes (PVDF), and blocked with 5% skim milk for 90 min at RT (Xiao et al., 2023). Then, the membranes were coincubated with the following primary antibodies overnight at 4°C: rabbit anti-Zona occludens protein 1 (ZO-1; Cat# 21773-1-AP; Proteintech Group, Inc.; 1:5,000, China); rabbit anti-Occludin (Cat# 27260-1-AP; Proteintech Group, Inc.; 1:2,000, China); and mouse anti-glyceraldehyde-3-phosphate dehydrogenase (GAPDH; Cat# 60004-1-Ig; Proteintech Group, Inc.; 1:5,000, China). After primary incubation, the membrane was washed with Tris-buffered-saline-Tween-20 (TBST) buffer three times for 10 min each and then incubated with horseradish peroxidase (HRP)-conjugated goat anti-rabbit secondary antibody (Cat#SA00001-2; Proteintech Group; 1: 5,000, China) or goat anti-mouse secondary antibody (Cat#SA00001-1; Proteintech Group; 1: 5,000, China) for 60 min at RT. Finally, the proteins were visualized using a super enhanced chemiluminescence reagent (ECL).

### 2.8 DNA extraction and 16S rRNA gene sequencing

For microbial DNA extraction, mouse feces samples were collected. Bacterial DNA was extracted from feces by the magnetic bead method combined with a genomic DNA kit (Cat# DP712-02; Tiangen Biotech Co., Ltd., China), and the purity and concentration of purified DNA were detected by spectrophotometry at 230 nm (A 230) and 260 nm (A 260). Then, the V4 region of the 16S ribosomal (r)RNA genes was amplified via primers (5’-AYTGGGYDTAAAGNG R-3’; 5’- TACNVGGGTATCTAATCC-3’) in the sample, and the PCR products were sequenced with an Illumina HiSeq 2000 platform (Illumina, Inc., San Diego, CA, USA) (GenBank accession number PRJNA1009232) (Zheng et al., 2021).

### 2.9 Proteomic analysis

Three biological replicate samples of *B. acidifaciens* (BA1, BA2, and BA3) and *B. acidifaciens*-derived EVs (EV1, EV2, and EV3) were obtained, and the 4D-label-free quantitative proteomic analysis was performed. The protein extraction of BA and EV was as previously described and quantified using the BCA protein assay kit. Protein digestion by trypsin was performed according to filter-aided sample preparation procedure described by Matthias Mann. LC-MS/MS analysis was performed on a timsTOF Pro mass that was coupled to Nanoelute (Bruker Daltonics). The MS raw data for each sample were combined and searched using the MaxQuant 1.5.3.17 software for identification and quantitation analysis (Chen et al., 2022; Zheng et al., 2023).

### 2.10 Statistical analysis

The data are represented by the mean standard deviation (SD). GraphPad Prism 9.0 (San Diego, CA, USA) was used for statistical analyses. And statistical analyses of two groups was performed by unpaired two-tailed Student’s *t*-test, multiple groups were performed by one-way ANOVA followed by Tukey’s multiple comparison test. The adjusted *P* < 0.05 was considered statistically significant (Zheng et al., 2021).

## 3 Results

### 3.1 The intervention of *B. acidifaciens* alleviated DSS-induced colitis

**FIGURE 1:**
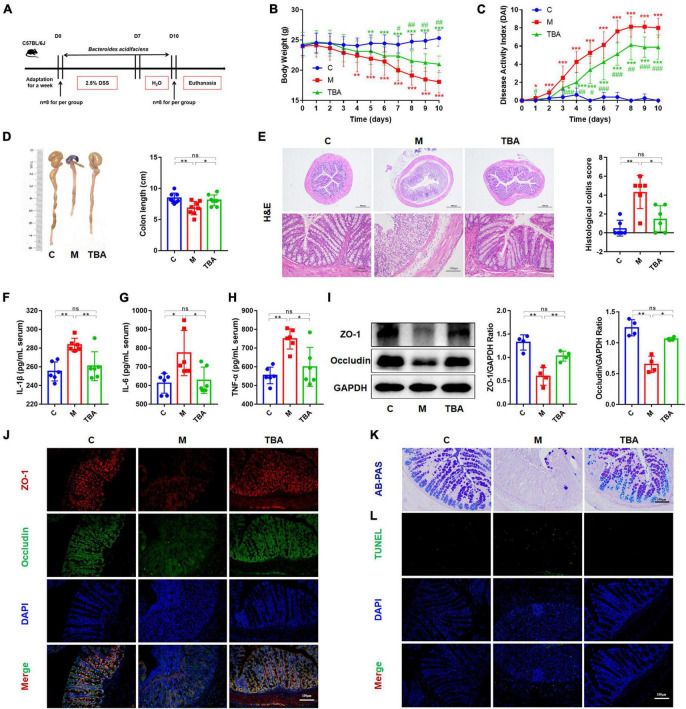
*Bacteroides acidifaciens* alleviated DSS-induced colitis in mice. **(A)** Experimental design. **(B)** Mouse body weight. **(C)** DAI score. **(D)** Colon length. **(E)** H&E staining and pathological score of colon sections. **(F–H)** Serum IL-1β, IL-6 and TNF-α levels. **(I)** The expression of tight junction proteins (ZO-1, Occludin) in mouse colon tissue. **(J)** Immunofluorescence was used to evaluate the expression of tight junction proteins (ZO-1, Occludin) in colonic sections. **(K)** Representative images of the Alcian blue-stained inner mucus layer of colonic sections. **(L)** Representative fluorescent pictures of TUNEL staining of colonic sections. C, control group; M, DSS group; TBA, DSS mice orally administered *B. acidifaciens* group. C, control group; M, DSS group; TBA, DSS mice orally administered *B. acidifaciens* group. Scale bar, 100 μm. Data are presented as the mean ± SD. *P < 0.05 versus the C group, # *P* < 0.05 versus the M group. ns, *P* > 0.05; */# *P* < 0.05; **/## *P* < 0.01; ***/### *P* < 0.001.

To observe the relieving effect of *B. acidifaciens* on colitis in mice, we used 2.5% DSS to induce colitis in mice for 7 days and treated them with *B. acidifaciens* at the same time for 10 days (Figure 1A). The results showed that compared with the M group, *B. acidifaciens* significantly alleviated weight loss, reduced the DAI and reversed the shortening of the colon (Figures 1B–D). Histological analysis revealed that inflammatory cells infiltrated the colon, intestinal villi were shortened and thickened, and mucosa was damaged in response to DSS treatment, while the administration of *B. acidifaciens* alleviated the colonic inflammatory reaction (Figure 1E). To further assess the effect of *B. acidifaciens* on the systemic inflammatory response, we detected proinflammatory cytokines in the serum of mice. The serum concentrations of IL-1β, IL-6 and TNF-α in *B. acidifaciens*-treated mice were significantly decreased (Figures 1F–H). To determine the protective effect of *B. acidifaciens* on the intestinal barrier, we further detected the expression of barrier proteins by western blot and immunofluorescence staining and detected mucin-secreting goblet cells in the colonic epithelium by AB-PAS staining. The results showed that oral administration of *B. acidifaciens* significantly reversed the decrease in barrier proteins caused by DSS (Figures 1I, J) and maintained better mucus distribution (Figure 1K). Moreover, TUNEL analysis further revealed that DSS significantly increased the frequency of apoptotic cells in the colonic mucosa, but *B. acidifaciens* largely reversed this trend (Figure 1L).

### 3.2 *B. acidifaciens* regulated the gut microbiota composition

**FIGURE 2:**
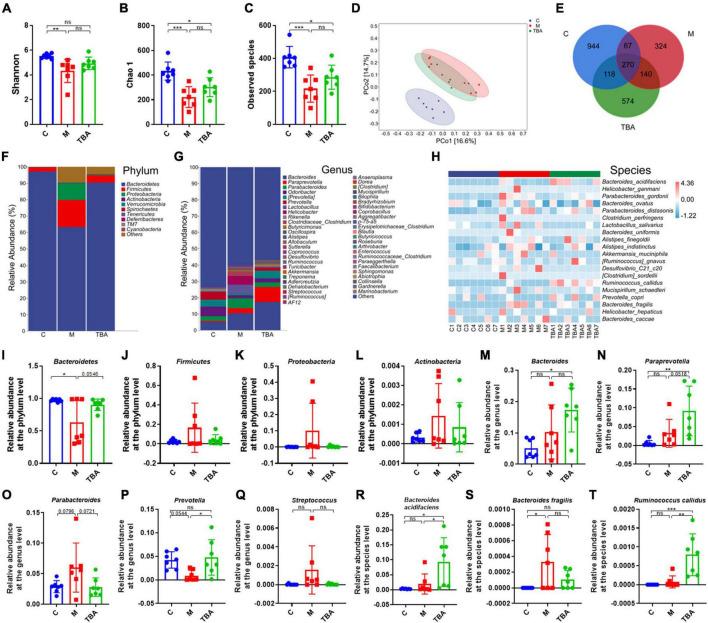
*Bacteroides acidifaciens* altered gut microbiota diversity in DSS-induced colitis mice. **(A)** Shannon index. **(B)** Chao 1 index. **(C)** Observed species. **(D)** PCoA of the β diversity index. **(E)** Venn diagram. **(F–H)** Relative abundance of the identified fecal microbiota at the **(F)** phylum, **(G)** genus, and **(H)** species levels as per 16S rRNA gene sequencing. **(I)** *Bacteroidetes*. **(J)** *Firmicutes*. **(K)** *Proteobacteria*. **(L)** *Actinobacteria*. **(M)** *Bacteroides*. **(N)** *Paraprevotella*. **(O)** *Parabacteroides*. **(P)** *Prevotella*. **(Q)** *Streptococcus*. **(R)** *B. acidifaciens*. **(S)** *Bacteroides fragilis*. **(T)** *Ruminococcus callidus*. C, control group; M, DSS group; TBA, DSS mice orally administered *B. acidifaciens* group. Data are presented as the mean ± SD. *n* = 7. *P < 0.05 versus the C group. ns, *P* > 0.05; *P < 0.05; **P < 0.01; ***P < 0.001.

Next, we further explored the influence of *B. acidifaciens* on the composition of gut microbiota in DSS-treated mice via 16S rRNA gene sequencing. The α-diversity analysis showed that DSS treatment significantly reduced the diversity of the gut microbiota in mice, and the administration of *B. acidifaciens* promoted its gradual recovery (Figures 2A–C). Principal coordinate analysis (PCoA) confirmed that DSS caused the gut microbiota to be located far away from the C group, while *B. acidifaciens* caused the TBA and C groups to be close to each other (Figure 2D). In addition, the Venn diagram showed (Figure 2E) that there were 1,399, 801, and 1,102 OTUs in the C, M and TBA groups, and the percentages of common OTUs in each group were 19.30% (270/1,399), 33.71% (270/801) and 24.50% (270/1,102), respectively.

Next, we further explored the gut microbiota composition and intestinal disease-related bacteria at the phylum, genus and species levels. At the phylum level, *Bacteroidetes*, *Firmicutes*, *Proteobacteria*, and *Actinobacteria* were predominant phyla in the fecal microbiota (Figures 2F, I–L). Moreover, the results showed that colitis mice showed a low relative abundance of *Bacteroides*, and the administration of *B. acidifaciens* reversed the relative abundance of *Bacteroides* (Figure 2I). At the genus level, the fecal microbiota was dominated by *Bacteroides*, *Paraprevotella*, and *Parabacteroides* (Figure 2G). Further analysis showed that the administration of *B. acidifaciens* significantly increased the relative abundance of *Bacteroides*, *Paraprevotella*, and *Prevotella* while decreasing the relative abundance of *Parabacteroides* and *Streptococcus* (Figures 2M–Q). The results of species level analysis showed that the administration of *B. acidifaciens* sharply increased the relative abundance of *B. acidifaciens* and *Ruminococcus callidus* but slightly decreased the relative abundance of *Bacteroides fragilis* (Figures 2H, R–T). However, there was no significant difference in the relative abundance of *B. fragilis* between the TBA group and the other two groups.

### 3.3 *B. acidifaciens*-changed gut microbiota and bacterial metabolites contributed to the alleviation of colitis

**FIGURE 3:**
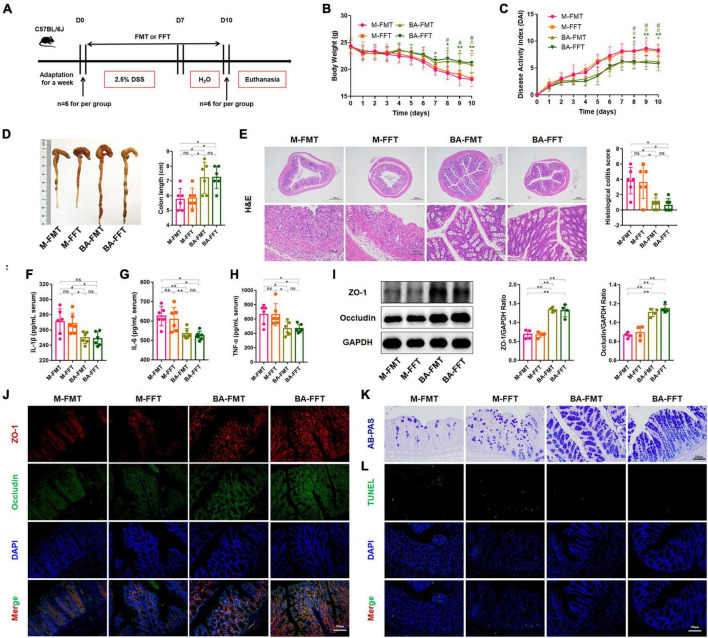
*Bacteroides acidifaciens* changed the gut microbiota, and metabolites contributed to alleviating colitis. **(A)** Experimental design. **(B)** Mouse body weight. **(C)** DAI score. **(D)** Colon length. **(E)** H&E staining and pathological score of colon sections. **(F–H)** Serum IL-1β, IL-6 and TNF-α levels. **(I)** The expression of tight junction proteins (ZO-1, Occludin) in mouse colon tissue. **(J)** Immunofluorescence representative images of tight junction proteins (ZO-1, Occludin) in colonic sections. **(K)** Representative images of the Alcian blue-stained inner mucus layer of colonic sections. **(L)** Representative fluorescent pictures of TUNEL staining of colonic sections. M-FMT: transplanted with fecal supernatant of the M group; M-FFT: transplanted with fecal supernatant filtrate of the M group; BA-FMT: transplanted with fecal supernatant of the TBA group; BA-FFT: transplanted with fecal supernatant filtrate of the TBA group. Scale bar, 100 μm. Data are presented as the mean ± SD. *P < 0.05 versus the M-FMT group, # *P* < 0.05 versus the M-FFT group. ns, *P* > 0.05; */# *P* < 0.05; **P < 0.01.

We further performed FMT and FFT to determine the role of gut microbiota and bacterial metabolites in the remission of colitis mediated by *B. acidifaciens* (Figure 3A). The results showed that compared with the FMT or FFT of the M group mice, the FMT and FFT of the donor mice treated with *B. acidifaciens* reduced the weight loss and DAI score and shortened the colon length (Figures 3B–D). Histological analysis showed that FMT or FFT of *B. acidifaciens*-treated donor mice significantly alleviated the infiltration of inflammatory cells into the colon, shortened and thickened villi, and damaged mucosa, thus reducing the histological score (Figure 3E). In addition, the FMT of *B. acidifaciens*-treated donor mice was helpful to reduce the level of serum inflammation, which was proven by the significantly reduced levels of IL-1β, IL-6 and TNF-α (Figures 3F–H). Furthermore, FMT and FFT of *B. acidifaciens*-treated donor mice significantly increased the expression of the barrier proteins ZO-1 and Occludin (Figures 3I, J) and maintained the distribution of intestinal mucus (Figure 3K). TUNEL analysis further revealed that FMT or FFT of *B. acidifaciens*-treated donor mice significantly reduced cell apoptosis in colon mucosa (Figure 3L). Collectively, *B. acidifaciens*-changed gut microbiota and metabolites were able to alleviate DSS-induced colitis.

### 3.4 *B. acidifaciens*-EV administration alleviated DSS-induced colitis

**FIGURE 4:**
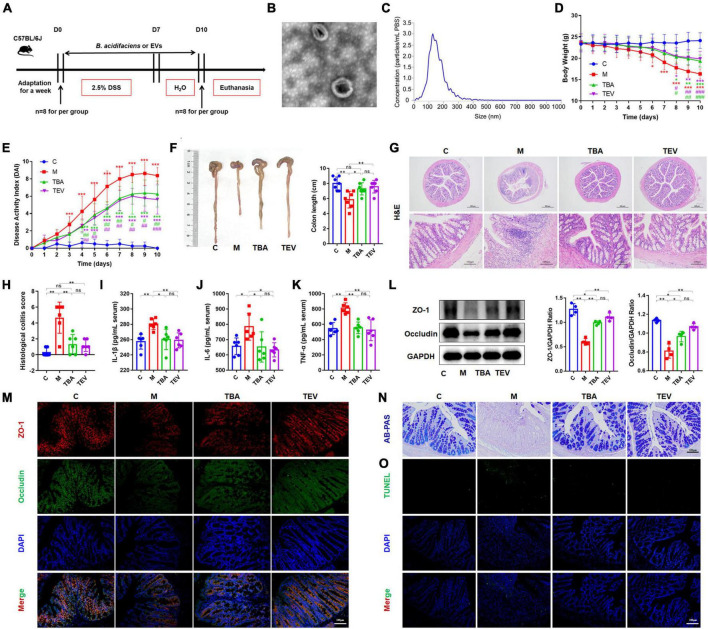
*Bacteroides acidifaciens*-EVs had a relieving effect on DSS-induced colitis. **(A)** Experimental design. **(B)** Observation of *B. acidifaciens*-EV morphology by means of TEM. Scale bar, 50 nm. **(C)** Observation of *B. acidifaciens*-EV size distribution analyzed by NTA. **(D)** Mouse body weight. **(E)** DAI score. **(F)** Colon length. **(G,H)** H&E staining and pathological score of colon sections. Scale bar, 100 μm. **(I–K)** Serum IL-1β, IL-6 and TNF-α levels. **(L)** The expression of tight junction proteins (ZO-1, Occludin) in mouse colon tissue. **(M)** Immunofluorescence representative images of tight junction proteins (ZO-1, Occludin) in colonic sections. **(N)** Representative images of the Alcian blue-stained inner mucus layer of colonic sections. **(O)** Representative fluorescent pictures of TUNEL staining of colonic sections. C, control group; M, DSS group; TBA, DSS mice orally administered *B. acidifaciens* group; TEV, DSS mice orally administered *B. acidifaciens*-EVs group. Data are presented as the mean ± SD. *P < 0.05 versus the C group, # *P* < 0.05 versus the M group. ns, *P* > 0.05; */# *P* < 0.05; **/## *P* < 0.01; ***/### *P* < 0.001.

Furthermore, we isolated and purified bacterial EVs to explore whether the EVs of *B. acidifaciens* alleviated DSS-induced colitis (Figure 4A). Transmission electron microscopy of EVs showed saucer-like vesicle morphology (Figure 4B). The NTA results showed that the diameter of the EVs was approximately 129 nm (Figure 4C). Furthermore, we explored whether *B. acidifaciens* EVs had a therapeutic effect on DSS-induced colitis. Compared with the M group, the administration of *B. acidifaciens* or its EVs resulted in lower body weight loss, DAI score, and shortened colon length (Figures 4D–F). In addition, we observed that the infiltration of inflammatory cells, tissue damage and histological score in the colon decreased after the administration of *B. acidifaciens* EVs (Figures 4G, H). Moreover, the levels of IL-1β, IL-6, and TNF-a were significantly reduced in the TBA and TEV groups (Figures 4I–K). Consistently, western blot and immunofluorescence analysis of ZO-1 and Occludin showed that *B. acidifaciens* or EV treatment weakened the epithelial tight junction protein barrier damaged by DSS (Figures 4L, M). The administration of *B. acidifaciens* or EVs also significantly enhanced the integrity of the mucus barrier, showing more mucin-positive coverage (Figure 4N). TUNEL analysis revealed that *B. acidifaciens* or EVs significantly reduced cell apoptosis in the colonic mucosa (Figure 4O). Notably, compared with the treatment of *B. acidifaciens*, EV administration can significantly alleviate the symptoms of acute colitis.

### 3.5 Proteomic analysis of *B. acidifaciens*-EVs and *B. acidifaciens*

**FIGURE 5:**
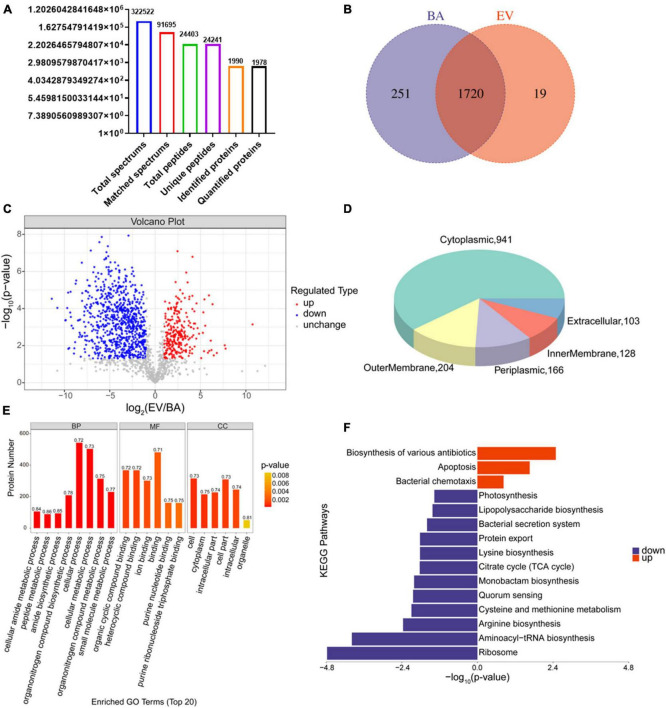
Proteomic analysis of *B. acidifaciens* -EVs and *B. acidifaciens*. **(A)** Summary of tandem mass spectrometry (MS/MS) database search results for *B. acidifaciens*-EVs and *B. acidifaciens*. **(B)** Venn diagram. **(C)** Volcano plot showing the number of proteins differentially expressed between *B. acidifaciens*-EVs and *B. acidifaciens*, with a cutoff of *P* < 0.05 and | fold change| > 2. **(D)** Subcellular localization pie chart of differentially expressed proteins in *B. acidifaciens*-EVs and *B. acidifaciens*. **(E)** GO classification (biological process, molecular function, and cellular components) of all differentially expressed proteins in *B. acidifaciens*-EVs and the top 20 enriched GO terms. **(F)** KEGG pathway enrichment analysis of the differentially expressed proteins in *B. acidifaciens*-EVs and *B. acidifaciens*. BA, *B. acidifaciens*; EV, *B. acidifaciens*-EVs. *n* = 3.

Proteins in *B. acidifaciens* EVs or *B. acidifaciens* were identified and quantified using 4D- label-free proteomic analysis. A total of 1,990 proteins were identified in *B. acidifaciens*-EVs and *B. acidifaciens*, of which 1,978 were quantified (Figure 5A). Venn diagram analysis showed that there were fewer unique differential proteins identified in EVs compared to *B. acidifaciens* (Figure 5B and Supplementary Table 1). Furthermore, we found that *B. acidifaciens* EVs resulted in 277 upregulated differentially expressed proteins (DEPs) and 788 downregulated DEPs (Figure 5C). As shown in Figure 5D, 61.02% of them were from cytoplasmic proteins, and 13.23% of them were annotated as outer membrane proteins. The subcellular localizations of 10.77% of the proteins were periplasmic, and the remaining proteins were from the inner membrane (8.30%) or extracellular (6.68%) (Figure 5D). To further identify biological alterations in *B. acidifaciens* EVs, the DEPs were classified into three categories based on gene ontology (GO) analysis: biological process (BP), molecular function (MF), cellular component (CC), and the top 20 terms were displayed in Figure 5E. The maximum proportion of DEPs was concentrated in bacterial biological processes, including cellular processes and cellular metabolic processes. Kyoto Encyclopedia of Genes and Genomes (KEGG) pathway enrichment analysis showed that the upregulated proteins in EVs compared with *B. acidifaciens* were involved in the biosynthesis of various antibiotics, apoptosis, and bacterial chemotaxis. Moreover, EVs downregulated proteins related to biosynthesis and metabolism, including lipopolysaccharide biosynthesis (Figure 5F). Thus, EVs underwent significant changes in protein composition compared to *B. acidifaciens*, and ultimately altering the biological functions.

## 4 Discussion

**FIGURE 6:**
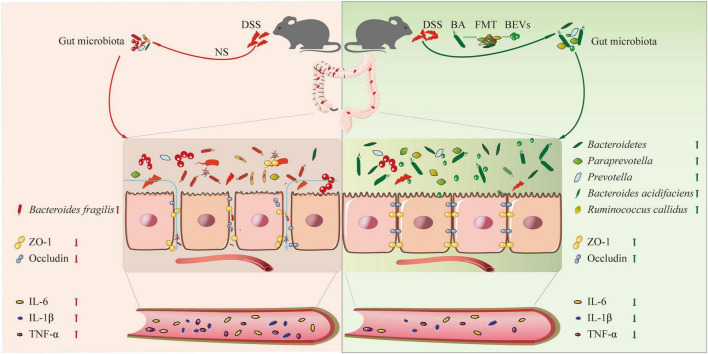
A schematic diagram summarizing the main findings of this study.

In this study, we explored whether *B. acidifaciens*, a specific intestinal symbiotic bacterium, can alleviate the symptoms of DSS-induced colitis by reducing the inflammatory reaction and repairing the intestinal mucosal barrier. In addition, through fecal transplantation and fecal filtrate transplantation from *B. acidifaciens*-treated mice, we confirmed that the gut microbiota and metabolites regulated by *B. acidifaciens* may be the reason the symptoms of colitis are alleviated. Finally, we proved that *B. acidifaciens* EVs can also alleviate the symptoms of colitis by reducing inflammatory reactions and repairing the intestinal mucosal barrier (Figure 6).

A previous study reported that oral administration of 3% DSS for 5 days can reduce EVs from *B. acidifaciens* and *Akkermansia muciniphila* (Paredes-Sabja et al., 2013). Another study revealed that the protective effect of sulfated polysaccharides from *Gracilaria lemaneiformis* on DSS-induced colitis in mice was closely related to probiotics such as *Alistipes* and *B. acidifaciens* (Han et al., 2021). To explore whether oral administration of *B. acidifaciens* can alleviate colitis, acute colitis in mice was induced by 2.5% DSS and then treated with oral *B. acidifaciens*. Consistent with previous studies, we confirmed that the therapeutic application of oral *B. acidifaciens* can alleviate DSS-induced colitis, as shown by the DAI score, colon length and histological damage (Hu et al., 2020; Dong et al., 2022; Sharma et al., 2023). Similarly, oral *B. acidifaciens* also alleviated colonic inflammation, as exemplified by the decline in inflammatory cytokines such as IL-1β, IL-6 and TNF-α in the serum of DSS-treated mice (Hao et al., 2021; Zhao et al., 2021; Li X. et al., 2022). Furthermore, oral administration of *B. acidifaciens* alleviated DSS-induced damage to integrity and barrier function, as evidenced by the increased colonic mucus as well as the enhanced tight junctions of the colonic mucosa, in agreement with previous studies (Cui et al., 2021; Wu H. et al., 2021). Moreover, DSS-induced colitis can lead to apoptosis of colon tissue cells, while oral administration of *B. acidifaciens* reduced this phenomenon (Qu et al., 2017; Li et al., 2019; Kim et al., 2023).

Furthermore, we investigated the differences between the feces of mice orally administered *B. acidifaciens* (TBA) and the other two groups (C and M). The results of α-diversity analysis showed that the DSS-induced group significantly reduced the microbial diversity of mouse feces, while supplementation with *B. acidifaciens* reversed this result (Zhang et al., 2019; Huang et al., 2022). PCoA revealed differences in the microbial community structure among the C, M and TBA groups, with the microbial community structure of the TBA group gradually approaching that of the C group. Further analysis of the gut microbiota composition of mice treated with *B. acidifaciens* showed a significant increase in the abundance of *Bacteroides*, *Paraprevotella*, and *Prevotella*. A previous study revealed a decrease in the metabolites of *Bacteroides* and SCFAs in IBD patients (Deleu et al., 2021; Guo et al., 2021; Zhang et al., 2022). The *Paraprevotella* genus has been proven to have anti-inflammatory effects (Renson et al., 2020). *Lachnospira* and *Paraprevotella* are symbiotic bacteria that may produce short-chain fatty acids (such as succinic acid or acetic acid) in the gastrointestinal tract (Hattori et al., 2020). In addition, *Paraprevotella* can accumulate at the healing site of the intestinal mucosa (Feng et al., 2021). Another study reported that the establishment of a UC model can reduce the proportion of *Prevotella* species, while using drugs to alleviate enteritis can restore their relative abundance (Liu et al., 2020). Specific species of the *Prevotella* genus can successfully inhibit inflammatory demyelinating disease and multiple sclerosis by inducing Tregs and regulating cytokines (Mangalam et al., 2017; Shahi et al., 2019). Moreover, supplementation with *B. acidifaciens* also reduced the relative abundance of *Parabacteroides* and *Streptococcus* (Li Q. et al., 2022; Wu et al., 2023). Fabio Cominelli et al. reported that *Parabacteroides distasonis* opportunistically colonizes the gut niche of susceptible CD patients and exerts a depressive effect (Gomez-Nguyen et al., 2021). At the species level, supplementation with *B. acidifaciens* significantly reduced the relative abundance of *B. fragilis*. Toshifumi Hibi et al. revealed that *B. fragilis* was the main bacterial species in the feces of UC patients (Takaishi et al., 2008). In the present study, oral administration of *B. acidifaciens* significantly increased the relative abundance of *Ruminococcus callidus*, which was negatively correlated with intestinal symptoms, especially discomfort (Hiel et al., 2019).

Previous studies have reported that disease treatment can be achieved by transplanting functional microflora (FMT) from healthy human feces into the gastrointestinal tract of patients with ulcerative colitis and rebuilding a new gut microbiota (Moayyedi et al., 2015; Sokol et al., 2020). Similarly, transplanting fecal filtrate (FFT) from treatment group mice to enteritis mice can alleviate the colitis phenotype by eliminating ecological imbalances (Wu Z. et al., 2021; Ma et al., 2022). To verify the effects of microbiota and metabolites mediated by *B. acidifaciens*, we performed FMT and FFT. Consistent with the treatment with *B. acidifaciens*, BA-FMT was more effective than M-FMT in alleviating the phenotype of colitis in mice, reducing inflammatory reactions and repairing the intestinal mucosal barrier (Dong et al., 2021). In addition, the treatment of FFT in donors with *B. acidifaciens* also alleviated DSS-induced colitis (Wu Z. et al., 2021). We demonstrated that the gut microbiota and its metabolites induced by *B. acidifaciens* played a key role in alleviating DSS-induced experimental colitis.

Bacterial EVs are an important bridge for information exchange between gut microbiota and host cells (Toyofuku et al., 2019). EVs selectively carry various molecules (such as nucleic acids and proteins) from their bacteria, which may regulate host signaling pathways and various cellular processes (Toyofuku et al., 2019; Díaz-Garrido et al., 2021). To explore the interaction between *B. acidifaciens* and the host, we further investigated the role of EVs derived from *B. acidifaciens* in preventing colitis. As expected, *B. acidifaciens* and its derived EVs improved colitis symptoms, including colitis phenotype and histological scores (Hao et al., 2021; Tong et al., 2021). Consistent with previous studies, EVs treatment also reduced the expression of proinflammatory genes, such as IL-1β, IL-6, and TNF-α (Hao et al., 2021; Tong et al., 2021). Moreover, EVs enhance the epithelial barrier by regulating the expression of the TJ proteins Occludin and ZO-1, stabilizing the distribution of intestinal mucin proteins, and reducing intestinal tissue cell apoptosis (Kang et al., 2020). In summary, the above results emphasize the crucial role of probiotic-derived EVs in regulating DSS-induced colitis.

Compared to bacteria themselves, EVs derived from bacteria are rich in various functional proteins that can directly act on receptor cells and perform corresponding functions (Brown et al., 2015). Previous studies have shown that compared to *Lactobacillus animalis*, *L. animalis* EVs may be able to transfer some pro-angiogenic, pro-osteogenic, and anti-apoptotic proteins to endothelial and bone cells to exert bone protective effects (Chen et al., 2022). Another study reported that signal exchange between the microbiota and host bone can effectively alleviate osteoporosis by transferring *Akkermansia muciniphila* functional EVs from bacteria to bone cells (Liu J. H. et al., 2021). Therefore, we further utilized proteomics analysis to investigate the differential expression of extracellular vesicular proteins derived from *B. acidifaciens*. Bacteria have evolved unique ways of killing each other, so many of our most powerful antibiotics are derived from the bacteria themselves (Niehus et al., 2021). Our research findings suggested that *B. acidifaciens*-EVs have enriched biosynthesis of various antimicrobial-related proteins, which may be their potential mechanism for inhibiting the growth of other bacteria (Domínguez Rubio et al., 2022; Ma et al., 2023). Lipopolysaccharides are components of the outer wall of gram-negative bacterial cells and are endotoxins that can exert their toxic effects by acting on animal cells (Wang and Quinn, 2010). Additionally, we observed that *B. acidifaciens*-EVs also downregulated biosynthesis- and metabolism-related proteins, including lipopolysaccharide biosynthesis, which may be an indirect mechanism of the action of eosinophils on DSS-induced colitis, which still requires future exploration.

## 5 Conclusion

In summary, *B. acidifaciens* and its derived EVs can alleviate DSS-induced colitis by reducing mucosal damage to colon tissue, reducing inflammatory response, promoting mucosal barrier repair, restoring gut microbiota diversity, and restoring gut microbiota balance in mice. In this study, we clarified the effect of *B. acidifaciens* on DSS-induced colitis and explored its mechanism of action through its derived EVs. And through proteomics, it was found that there are differences in the proteomics of *B. acidifaciens* and its EVs. However, how EVs communicates with the host and participates in host regulation may be the key to the treatment of colitis with *B. acidifaciens*, and further exploration is needed. The results of this study provide a theoretical basis for the preclinical application of the new generation of probiotics.

## Data availability statement

The datasets presented in this study can be found in online repositories. The names of the repository/repositories and accession number(s) can be found below: NCBI–PRJNA1009232.

## Ethics statement

The animal study was approved by the Animal Experiment Ethics Committee of Nanchang University (NCULAE-202210311149). The study was conducted in accordance with the local legislation and institutional requirements.

## Author contributions

CZ: Methodology, Visualization, Writing – original draft. YZ: Methodology, Software, Visualization, Writing – original draft. JX: Data curation, Methodology, Software, Writing – original draft. ZW: Methodology, Software, Writing – original draft. WenmZ: Methodology, Visualization, Writing – original draft. YP: Methodology, Writing – original draft. WenjZ: Visualization, Writing – original draft. LL: Methodology, Writing – original draft. JL: Conceptualization, Funding acquisition, Writing – original draft. WX: Conceptualization, Funding acquisition, Methodology, Writing – original draft, Writing – review & editing.
